# Real-World Evidence Study of Patients with *KRAS*-Mutated NSCLC in Finland

**DOI:** 10.3390/curroncol31050205

**Published:** 2024-05-11

**Authors:** Anna Anttalainen, Paavo Pietarinen, Samuli Tuominen, Riikka Mattila, Aino Mutka, Aija Knuuttila

**Affiliations:** 1 Medaffcon Oy, 02130 Espoo, Finland; 2 Amgen AB, 02150 Espoo, Finland; 3 Department of Pathology, University of Helsinki and Helsinki University Hospital, 00290 Helsinki, Finland; 4 Department of Pulmonary Medicine, Heart and Lung Center and Cancer Center, Helsinki University Hospital, 00290 Helsinki, Finland

**Keywords:** lung cancer, NSCLC, KRAS, real-world evidence

## Abstract

While *KRAS* is the most frequently mutated oncogene in non-small cell lung cancer (NSCLC), *KRAS*-mutant tumors have long been considered difficult to treat and thus, an unmet need still remains. Partly due to the lack of targeted treatments, comprehensive real-world description of NSCLC patients with *KRAS* mutation is still largely missing in Finland. In this study, all adult patients diagnosed with locally advanced and unresectable or metastatic NSCLC from 1 January 2018 to 31 August 2020 at the Hospital District of Helsinki and Uusimaa were first identified in this retrospective registry-based real-world study. The final cohort included only patients tested with next generation sequencing (NGS) and was stratified by the *KRAS* mutation status. A total of 383 patients with locally advanced and unresectable or metastatic NSCLC and with NGS testing performed were identified. Patients with *KRAS* mutation (*KRAS* G12C *n* = 35, other *KRAS n* = 74) were younger than patients without *KRAS* mutations, were all previous or current smokers, and had more often metastatic disease at diagnosis. Also, these patients had poorer survival, with higher age, Charlson comorbidity index (CCI) being 5 or above, and *KRAS* G12C being the most significant risk factors associated with poorer survival. This suggests that the patients with *KRAS* mutation have a more aggressive disease and/or tumors with *KRAS* mutation are more difficult to treat, at least without effective targeted therapies.

## 1. Introduction

Non-small cell lung cancer (NSCLC) is the most common lung cancer group, accounting for approximately 80–85% of all cases [1,2]. NSCLC patients have a poor prognosis, as they are frequently diagnosed at an advanced stage. Additionally, resistance to approved therapies underscores the need for more treatment options [3]. While smoking is a major risk factor for developing lung cancer, genetic alterations have been increasingly shown to describe the disease etiology. *KRAS* is the most frequently mutated oncogene in NSCLC, especially found in adenocarcinomas in 20–30% of cases [4,5,6]. *KRAS* mutations, particularly G12C, are typically found in patients with a history of heavy smoking [7].

The treatment of NSCLC has improved drastically in recent years with the introduction of new personalized and immuno-oncological treatment options. Molecular genetic analysis has become a routine procedure in diagnostics and guiding treatment decision making, especially for non-squamous cell carcinomas. Several alterations, such as those found in EGFR, ALK, ROS1, NTRK, MET, RET, BRAF V600, and HER2, already have targeted therapies, and more are coming. This has led to an improvement in life expectancy for many of these patients, also in advanced stages [8], although long-term outcomes still often remain poor.

In some studies, lung cancers with *KRAS* mutations have been associated with inferior overall survival (OS) compared to *KRAS* wild-type tumors, especially in the advanced stages [9,10], although the evidence is still inconclusive [11]. Due to the complex mutational profile, *KRAS*-mutant tumors have long been considered difficult to treat with targeted therapy. Today, there are some KRAS^G12C^ targeting therapies (sotorasib and adagrasib) [12,13,14], but an unmet need still remains, and their reimbursement and availability differ between countries.

The total economic impact of lung cancer is significant, being responsible for the highest estimated cost among all cancers [15]. In Europe, the cost of lung cancer care is estimated as EUR 18.8 billion each year, corresponding to 15% of the total cancer-related costs [16]. When the costs associated with disability and premature mortality are also included, the total annual costs equal to over EUR 100 billion [17].

No registry data exist on real-world outcomes or treatment of patients with *KRAS*-mutated NSCLC in Finland. This retrospective real-world study aims to answer the need to understand the outcomes and healthcare resource utilization (HCRU) of patients with locally advanced and unresectable or metastatic NSCLC stratified by the *KRAS* status.

## 2. Materials and Methods

This was a retrospective registry-based real-world study at the Hospital District of Helsinki and Uusimaa (HUS) in Finland. The inclusion period was from 1 January 2018 to 31 August 2020 and the patients were followed until 28 February 2021. Data were collected from electronic health records (EHR) of HUS data lake, and were supplemented with demographics data from the Finnish Digital and Population Data Services Agency. This study was approved by the Finnish Social and Health Data Permit Authority Findata (THL/5970/14.02.00/2020). According to the Finnish law on secondary use of health and social data (552/2019), no patient consent was required. All analyses were performed using R version 4.0.3, Austria [18].

### 2.1. Cohort Formation

**Figure 1:**
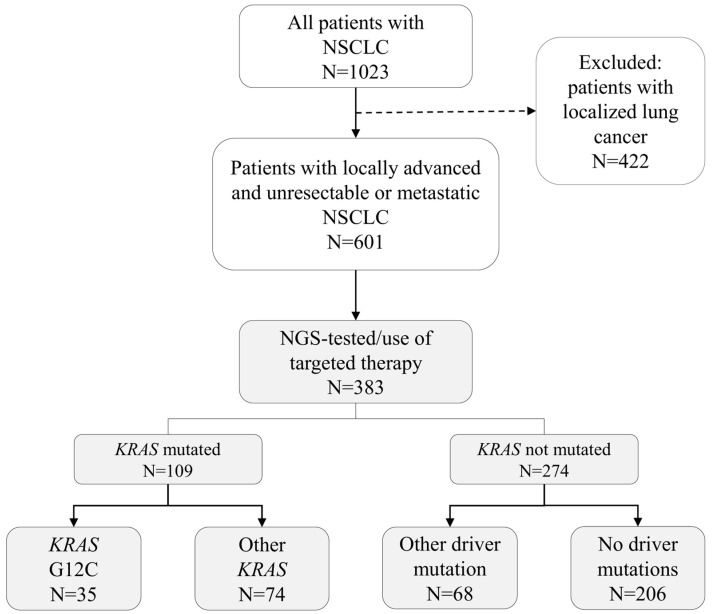
Flow chart of the cohort formation and patient stratification.

All adult (≥18 years of age) patients with NSCLC living in HUS area at diagnosis were included in the preliminary NSCLC cohort (Figure 1) using ICD-10 diagnosis codes and mSNOMED-codes (Supplementary Materials). Only patients with first ICD-10 or mSNOMED record related to NSCLC from 1 January 2018 to 31 August 2020 were considered, i.e., newly diagnosed patients. Patients with small-cell lung cancer at any time point were excluded. From the preliminary cohort, locally advanced and unresectable or metastatic patients were identified based on 5 criteria: ICD10-code (C77, C78, C79), radiation therapy of metastasis (WF049, AAG50), TNM-status (T4, N1-N3, M1), stage III–IV, and metastasis mentioned in the EHRs with free-form text. If any of the criteria was met during the time period of 3 months before the NSCLC diagnosis until the end of follow-up (death or end of study, i.e., 28 February 2021, whichever occurred first), the patient was considered locally advanced or metastatic. These criteria were required to be fulfilled after possible lung surgery/resection to exclude resectable patients. Only patients with locally advanced and unresectable or metastatic NSCLC and with successfully performed next generation sequencing (NGS) gene testing performed earliest at 3 months prior to NSCLC diagnosis and at latest by the end of follow-up were included in the final cohort.

The date of the first mention of locally advanced cancer or metastasis (any of the criteria listed above) was set as the index date for the advanced or metastatic NSCLC. Patients were followed from index until death or end of study period, whichever occurred first.

### 2.2. Patient Stratification

Patients were stratified into 4 subgroups based on NGS test results and medications: *KRAS* G12C, other *KRAS* mutation (i.e., other than G12C), other driver mutation (i.e., driver mutation in *EGFR*, *ALK*, *BRAF*, *MET*, *RET*, *ROS1*, *NTRK*, or *HER2* and no *KRAS* mutations), and no driver mutations (wild type to all abovementioned genes). If multiple tests were performed, only the latest results were used. As gene fusion test results were not available for this study, the presence of gene fusions was deduced from use of targeted medications for ALK, ROS1, RET, and NTRK between the time period of 3 months before index to end of follow-up.

### 2.3. Patient Characteristics

Patients were characterized at index and only records in a ±3-month time-window around the index were considered. Charlson comorbidity index (CCI) was calculated based on co-diagnoses recorded at baseline, at most 2 years prior to the index. The CCI was calculated using a scoring system developed by Quan et al. [19]. NSCLC (C34*) and metastasis (C77, C78, C79) diagnoses were not considered when calculating the CCI to avoid biases.

For continuous variables, median and interquartile range (IQR) were reported. For categorial variables, the number and percentage of patients for each class were reported. Number of patients in each category was compared to the number of patients for whom the respective data were available in the assessed cohort/subgroup, while the number of patients with missing values was compared to the total number of patients. The difference between strata was tested using Kruskal–Wallis test for the continuous variables and Chi-squared/Fisher’s exact test for the categorical variables. Significance level of 0.05 was used throughout the analyses. *p* values were not adjusted for multiple testing.

### 2.4. Treatment Lines

Data of treatments consisted of all drug administrations and prescriptions with ATC-code starting with L01 as well as radiation therapy from procedure data. Treatment lines were therefore constructed post hoc and were considered to change mainly when the active substance changed. However, at maximum a gap of 90 days for intravenous (IV) medications and 150 days for per oral (PO) medications was allowed within a treatment line to consider it as one continuous line of treatment.

### 2.5. Treatment Outcomes

Overall survival was analyzed using Kaplan–Meier fit with 95% CIs as time from index until death (event) or end of study (28 February 2021; censoring event). Additionally, the association of patient characteristics with OS was assessed using Cox proportional hazards model. The proportional hazards assumptions were checked. 

Time to next treatment (TTNT) was analyzed for the first treatment line using a competing risk model as time from the start of the first treatment line until the start of second treatment line (event), death (competing event), or end of study (censoring event). Median TTNT was defined from a Kaplan–Meier fit with start of the second treatment line and death as a composite event.

### 2.6. Healthcare Resource Utilization

All-cause healthcare resource utilization (HCRU) was assessed from the follow-up using data of outpatient visits (including emergency room visits), hospitalizations, and inpatient days. The corresponding costs were evaluated using unit costs (comprising the cost of the visit/stay, an average cost of performed procedures, operations and laboratory tests, and an average cost of given medications per specialty) [20]. For each contact type, the total number of contacts and corresponding costs were scaled to per patient (PP) and per patient month (PPM) estimates dividing the total estimates with number of patients and number of patient months, respectively. The 95% confidence intervals (CIs) were estimated using bootstrapping over the patients (10,000 bootstrap samples). 

## 3. Results

### 3.1. Patient Characteristics and Treatments

A total of 1023 patients with NSCLC were identified and of these 601 were at diagnosis or became locally advanced and unresectable or metastatic during the study period (Figure 1). A total of 383 patients (64%; 90% in adenocarcinomas and 19% in other NSCLCs) were tested for genetic alterations with NGS and of these 35 (9%) had the *KRAS* G12C mutation, 74 (19%) other *KRAS* mutation (Gly12Val; *n* = 28, Gly12Asp; *n* = 15, Gly12Ala; *n* = 11, Gly61His; *n* = 8, Gly12Ser; *n* < 5, Gly13Cys; *n* < 5, Gly12Arg; *n* < 5, Ala146Thr; *n* < 5, Gln61Leu; *n* < 5, Gln61Lys; *n* < 5, Gly12Phe; *n* < 5, Gly13Asp; *n* < 5), 68 (18%) other driver mutation (*BRAF*; *n* = 14, *EGFR*; *n* = 40, *MET* or *HER2*; *n* = 14), and 206 (54%) did not have any driver mutations detected. Median time from index to NGS sample was −0.5 months (IQR −1.8–0.13) meaning that the NGS sample was taken before the diagnosis of locally advanced or metastatic disease for at least half of the patients.

At diagnosis, patients with any *KRAS* mutation were younger than patients with other driver mutations (median age of 68 years in *KRAS* G12C and other *KRAS* subgroups, whereas median age of 72 years in other driver mutation subgroup; *p* = 0.033 Table 1). All patients with any *KRAS* mutation were previous or current smokers while a few patients with (*n* = 5, 8%) or without (*n* = 10, 5%) other driver mutations included some never-smoked patients (*p* < 0.001). Also, the cancer was already metastatic (stage IV) at diagnosis somewhat more often in patients with *KRAS* mutation than in other patient groups (*KRAS* G12C *n* = 12, 57%; other *KRAS n* = 31, 61%; other driver mutation *n* = 22, 43%; no driver mutations *n* = 61, 44%) indicating more aggressive disease.

In total, 213 patients (55.6%) received first-line therapy (Table 1): 42 patients (19.7%) received immune-oncological (IO) therapy either as monotherapy or in combination with chemotherapy (92.3% being platinum-based chemotherapy), 40 (18.8%) received tyrosine kinase inhibitors with or without platinum-based chemotherapy, 115 (54.0%) received platinum-based chemotherapy, and 16 (7.5%) received other chemotherapy. From these patients, only 81 (38.0%; *KRAS* G12C *n* < 5; other *KRAS n* = 19, other driver mutation *n* = 11, no driver mutation *n* = 47) patients reached a second treatment line during the study period. Only 28 patients reached a third treatment line (13.1%; other *KRAS n* = 6, other driver mutation *n* = 6, no driver mutations *n* = 16). None of the patients with the *KRAS* G12C mutation reached a third treatment line. In total, 170 patients (44.4%) received palliative/best supportive care (BSC) only (*n* = 63 BSC only, *n* = 107 BSC with radiotherapy).

### 3.2. Overall Survival and Time to Next Treatment

**Figure 2:**
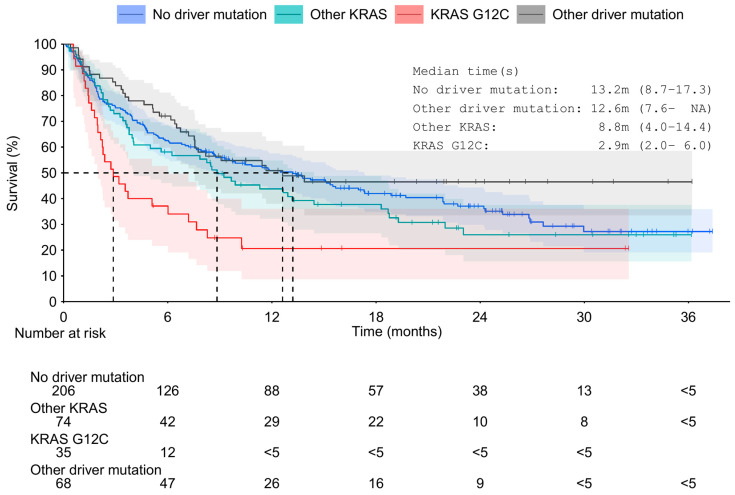
Overall survival of advanced and unresectable or metastatic NSCLC patients from diagnosis of advanced or metastatic disease stratified by *KRAS* status. Median OS: *KRAS* G12C 2.9 months (95% CI 2.0–6.0), other *KRAS* 8.8 months (95% CI 4.0–14.4), other driver mutation 12.6 months (95% CI 7.6–NR), no driver mutations 13.2 months (95% CI 8.7–17.3). NR = not reached. CI = confidence interval.

**Figure 3:**
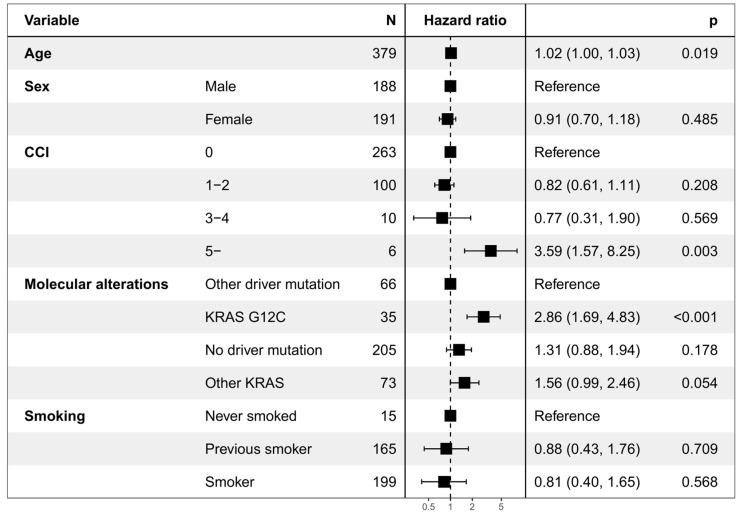
Cox model for OS. Hazard ratio above 1 indicates poorer and below 1 better survival compared to reference.

In general, OS was shorter for patients with *KRAS* mutation than for patients with or without other driver mutations (*p* = 0.002; Figure 2). The shortest median OS of 2.9 months (95% CI 2.0–6.0) was observed for patients with the *KRAS* G12C mutation, and the longest median OS of 13.2 months (95% CI 8.7–17.3) for patients without any driver mutation. Based on the Cox proportional hazards model (Figure 3), higher age, CCI 5 or above, and *KRAS* G12C status were significantly associated with the survival probability. Each additional 10 years in age increased the risk of death by a factor of 1.22 (HR for each additional year being 1.02, 95% CI 1.00–1.03, *p* = 0.019) and *KRAS* G12C mutation by a factor of 2.86 (95% CI 1.69–4.83, *p* < 0.001) compared to patients with a driver mutation in another gene. Patients with unknown smoking status (1%) were excluded from the model. When adjusting the model also by treatment status, performance status, and tumor stage (Supplemental Figure S1), the HR for *KRAS* G12C was slightly lower (1.75, 95% CI 1.01–3.04) but still significant (*p* = 0.048).

From the start of the first treatment, the median event-free survival (i.e., time to start of the second line or death) was 2.8 months (95% CI 1.2–5.2) for the patients with the *KRAS* G12C mutation, 6.9 months (95% CI 3.4–10.4) for the patients with other *KRAS* mutation, 10.1 months (95% CI 5.7–16.5) for the patients with other driver mutation, and 7.9 months (95% CI 6.3–11.1) for the patients without any driver mutations (Supplemental Figure S2).

### 3.3. Healthcare Resource Utilization

**Figure 4:**
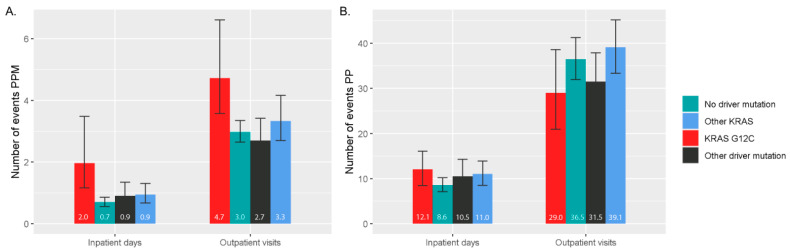
Healthcare resource utilization per patient month (**A**) and per patient (**B**) as number of outpatient visits, hospitalizations, and inpatient days.

On average, patients with locally advanced and unresectable or metastatic NSCLC had three to five outpatient visits and spent 1–2 days hospitalized PPM (Figure 4A). The PPM HCRU was somewhat higher for patients with *KRAS* G12C than for the other subgroups. The related costs were EUR 2600 PPM (95% CI EUR 1800–3900) for patients with *KRAS* G12C, EUR 1600 PPM (95% CI EUR 1300–2100) for patients with other *KRAS* mutation, EUR 1500 PPM (95% CI EUR 1100–2000) for patients with other driver mutation, and EUR 1400 PPM (95% CI EUR 1200–1600) for patients without any driver mutations. On the other hand, the per patient HCRU (total from index to death or EOF) varied less between the subgroups (Figure 4B) being 30–40 outpatient visits and two hospitalization periods lasting in total 8–12 days.

## 4. Discussion

This study, being the first one in Finland, described the real-world characteristics, overall survival, and healthcare resource utilization of Finnish locally advanced and unresectable or metastatic NCSLC patients by *KRAS* mutation status. Some studies have recently examined the real-world treatments and outcomes of NSCLC patients in Finland [21,22,23], but no analyses on patients with *KRAS* mutation and their survival have been published. In the current study, altogether 601 patients with locally advanced and unresectable or metastatic NSCLC were identified, out of which 383 were tested with NGS. Out of the 109 patients who were found positive for *KRAS* mutation, 35 patients (32.1%) were positive for the *KRAS* G12C mutation which is roughly in line with previous findings of *KRAS* G12C frequency [6,23,24]. Almost half (46%) of the patients had some driver mutation (*KRAS* or other driver mutation) that may affect further diagnostics and treatment options emphasizing the need for early NGS testing in NSCLC.

In general, the baseline characteristics and outcomes of this study were in line with previous studies [8,21,22]. While lung cancer has long been more frequent in men [25], here the sex distribution was equal in other groups except in the group of other driver mutations, where 65% were females as expected. However, our results reasonably agree with previously reported numbers from Finland [26], indicating a narrowing gap between genders. Additionally, all patients with any *KRAS* mutation were identified as smokers (previous or current) agreeing with previously found association of a substantial history of smoking and *KRAS*-mutant tumors [7].

In our study, we observed that patients with the *KRAS* G12C mutation exhibited significantly poorer survival compared to other patient groups suggesting a more aggressive disease and/or difficulty to treat the tumor with complex mutational landscape, at least without effective targeted therapies. Notably, a higher proportion of patients in the *KRAS* G12C group receiving BSC/palliative care only may partly explain the observed survival differences. Interestingly, similar RWE studies in Sweden [27], Denmark [24], and Germany [28] observed no or more modest decline in the survival in patients with *KRAS* G12C. For example, Frost et al. [24] reported a median survival of 7.1 months for patients with *KRAS* G12C, 7.3 months for patients with any *KRAS* mutations, and 7.3 months for patients with wild-type *KRAS*, *EGFR*, and *ALK*. Also, Frost et al. reported that in all subgroups, more than 60% of patients received first-line therapy, while in our study only in subgroups of other *KRAS* mutations and other driver mutations the proportion of patients receiving first-line therapy was over 60%. Among patients with *KRAS* G12C, less than half of the patients received first-line therapy in our study. However, differences in inclusion criteria and subgroup definitions in other RWE studies make the direct outcome comparison difficult.

Also, this study was timed during an era predating the availability of novel therapies, IO drugs. Consequently, patients were mostly treated with traditional chemotherapies, which may have also influenced the outcomes to be relatively poor compared to other studies. More timely investigations are needed in Finland to be able to include IO therapies and assess their impact on survival.

Furthermore, only incident NSCLC patients whose disease was or progressed to advanced or metastatic during 1 January 2018 to 31 August 2020 were included in our study. This inadvertently excluded patients with slower disease progression, whose disease advanced after our inclusion period. This may have led to overrepresentation of patients with more aggressive disease (i.e., advanced or metastatic disease already at the diagnosis of NSCLC or early relapse). This and including both treated patients and patients with BSC only may explain the relatively short median OS of 2.9 months in the *KRAS* G12C group compared to other studies, e.g., [27,28,29]. More studies with larger cohort sizes (to be able to separate treated patients and patients with BSC only in outcome analyses) and aiming to include also later relapses are needed to assess these aspects. 

In this study, patients with locally advanced and unresectable or metastatic NSCLC used more healthcare resources than an average specialty care patient in Finland in 2020 and 2021. Each month, patients with NSCLC had three to five outpatient visits and spent 1–2 days hospitalized compared to three outpatient visits and 8 inpatient days for an average specialty care patient during the whole year of 2020 [30]. The resulting PPM costs of the NSCLC cohort were EUR 1400–2600 compared to the specialty care costs of EUR 140–180 of an average 70-year-old Finnish inhabitant in 2020 (index correction and scaling to PPM estimates performed to the costs presented in Kapiainen et al.) [31]. HCRU is largely dependent on the disease progression, especially brain metastasis [32], and therefore varies largely between patients with NSCLC. When considering PPM HCRU estimates, the *KRAS* G12C patient group stands out from the other patient groups with somewhat higher HCRU potentially due to more aggressive disease. PP HCRU was similar across all subgroups, as the patients with the *KRAS* G12C mutation possessed significantly shorter survival and therefore, shorter follow-up to accumulate PP costs.

The current study was based on existing real-world data (RWD) from healthcare registers and involved automatic and algorithmic data extraction and processing and thus, laborious and slow chart review was not needed. Most of the analyzed data were in a structured format with high coverage. Only metastases, ECOG, and smoking status were text-mined from free-form text. However, data recorded in EHRs are subject to errors and incomplete records. Due to potential missing data, some locally advanced or metastatic NSCLC patients might not be captured in this study. The prevalence of central nervous system (CNS)/brain metastases is most likely underestimated here, as no standard screening (brain CT/MRI) was done for patients without symptoms of CNS metastasis. Also, the patient stratification was based on performed NGS tests and used targeted therapies and therefore reflected real-world testing practices, and not the objective mutational burden in NSCLC. Gene fusion test results were not available, and gene fusions were deduced from targeted therapies, leading to possible misclassification of gene fusion patients and an immortal survival bias until initiation of the treatment. However, <5 patients had gene fusion and therefore, the effect of the immortal survival bias is expected to be minimal. The co-occurring mutations were not analyzed in this study. Also, the number of patients was low in some patient subgroups, resulting in poorly defined or wide CIs. In groups where *n* < 5, the exact numbers of patients cannot be reported due to data privacy legislation, and thus some results have been censored or expressed as ranges rather than exact numbers. The results can be generalized to countries with similar population, healthcare system, and treatment practices.

## 5. Conclusions

Lung cancer is at the forefront of personalized medicine with genetic tests and targeted medications in routine use, especially for NSCLC patients with adenocarcinoma [33]. This field is developing rapidly, with new research findings, new genetic factors, and new targeted drugs, as well as immuno-oncology therapies coming to the market in rapid succession. This is expected to lead to better outcomes for the patients, but also to a very complex and continually evolving treatment landscape. This emphasizes the need for understanding the subgroups of patients with NSCLC and their outcomes in a real-world setting. 

## Figures and Tables

**Table 1:** Characteristics at index.

	Estimate/Level	Overall	*KRAS* G12C	Other *KRAS*	Other Driver Mutation	No Driver Mutation	*p* Value	Missing % ^b^
*n*		383	35	74	68	206		
Age in years	median [IQR]	69.0 [62.0, 75.0]	68.0 [59.5, 72.0]	67.5 [61.0, 73.0]	72.0 [66.0, 77.0]	69.0 [61.0, 74.0]	0.033	0
Age group, years, N (%)	≤59	83 (21.7)	8–11	13–16	8 (11.8)	51 (24.8)	0.143	0
	60–79	254 (66.3)	23 (65.7)	51 (68.9)	47 (69.1)	133 (64.6)		
	≥80	46 (12.0)	<5	7–10	13 (19.1)	22 (10.7)		
Sex, N (%)	Female	193 (50.4)	18 (51.4)	33 (44.6)	44 (64.7)	98 (47.6)	0.065	0
	Male	190 (49.6)	17 (48.6)	41 (55.4)	24 (35.3)	108 (52.4)		
Length of follow-up in days	median [IQR]	257.0 [87.5, 560.0]	87.0 [51.5, 242.5]	259.0 [83.5, 580.0]	263.5 [156.8, 474.8]	284.0 [101.0, 590.8]	0.004	0
Smoking status, N (%)	Never smoked	15 (4.0)	0 (0.0)	0 (0.0)	5 (7.6)	10 (4.9)	<0.001	1
	Previous smoker ^a^	165 (43.5)	8 (22.9)	27 (37.0)	40 (60.6)	90 (43.9)		
	Smoker	199 (52.5)	27 (77.1)	46 (63.0)	21 (31.8)	105 (51.2)		
Performance status, ECOG, N (%)	0–1	173 (66.5)	14 (77.8)	32 (66.7)	35 (71.4)	92 (63.4)	0.543	32.1
	2	52 (20.0)	<5	9 (18.8)	7–10	35 (24.1)		
	3–4	35 (13.5)	<5	7 (14.6)	5–8	18 (12.4)		
CCI score, N (%)	0	265 (69.2)	21 (60.0)	54 (73.0)	49 (71.1)	141 (68.4)	0.752	0.0
	1–2	102 (28.5)	10–13	16–19	15–18	55 (29.1)		
	3+	16 (4.5)	<5	<5	<5	10 (5.3)		
Histology, N (%)	Adenocarcinoma	340 (93.9	31–34	70–73	64–67	170 (90.4)	0.025	5.5
	Other NSCLC	22 (6.1)	<5	<5	<5	18 (9.6)		
Stage, N (%)	IA	<5	0 (0.0)	<5	0 (0.0)	<5	0.114	31.9
	IB	<5	<5	0 (0.0)	0 (0.0)	0 (0.0)		
	IIA	15 (5.7)	<5	0 (0.0)	<6	6–9		
	IIB	8 (3.1)	0 (0.0)	<5	<5	6 (4.3)		
	IIIA	73 (28.0)	5 (23.8)	10 (19.6)	16 (31.4)	42 (30.4)		
	IIIB	35 (13.4)	<5	8 (15.7)	7 (13.7)	19 (13.8)		
	IV	126 (48.3)	12 (57.1)	31 (60.8)	22 (43.1)	61 (44.2)		
Metastasis index delay in days	median [IQR]	7.0 [−4.0, 51.5]	1.0 [−7.5, 26.0]	2.0 [−9.8, 14.0]	10.0 [−6.3, 49.3]	15.5 [−1.0, 79.5]	<0.001	0
Brain metastasis, N (%)	Yes	59 (15.4)	<5	14 (18.9)	7–10	34 (16.5)	0.417	0
	No	324 (84.6)	31–34	60 (81.1)	58–61	172 (83.5)		
Received first-line therapy	Yes	213 (55.6)	17 (48.6)	48 (64.9)	42 (61.8)	106 (51.5)	0.124	0

^a^ Contains patients with any history of smoking in the past. ^b^ Missing percentages were equally distributed across strata (Fisher’s exact test *p* > 0.05), except for histology (*KRAS* G12C 2.9%, other *KRAS* 2.9%, other driver mutation 0%, no driver mutations 8.7%; *p* = 0.016). Some results are reported as ranges instead of exact number of patients, so that *n* < 5 results could not be deduced from other numbers as instructed by the Finnish authority Findata.

## Data Availability

This study is based on secondary use of healthcare register data. Hence, data cannot be shared openly.

## Supplementary Materials

The following supporting information can be downloaded at: https://www.mdpi.com/article/10.3390/curroncol31050205/s1, Figure S1: Cox model for OS including also treatment status, performance status, and tumor stage as covariates. The proportional hazard assumptions were violated for these additional covariates and thus, the hazard ratios represent an average risk over time. Figure S2: Time to next treatment of the first treatment line by *KRAS* subgroups using a competing risk model (death as competing risk). Event-free survival (blue curve) indicates the composite event of start of the next treatment line and death. Median TTNT from the event-free survival: *KRAS* G12C 2.8 months (95% CI 1.2–5.2), other *KRAS* 6.9 months (95% CI 3.4–10.4), other driver mutation 10.1 months (95% CI 5.7–16.5), no driver mutations 7.9 months (95% CI 6.3–11.1).
