# Supplementary material for: Real-World Evidence Study of Patients with KRAS-Mutated NSCLC in Finland
Source: Curr Oncol. 2024 May 11;31(5):2700–12. doi: 10.3390/curroncol31050205 (PMC11120216; doi:10.3390/curroncol31050205)
Supplement: Supplementary file 1 [file curroncol-31-00205-s001.zip › Supplemental material.pdf]

Supplemental material

ICD-10 codes for NSCLC: C34.01- C34.05, C34.09, C34.11-C34.15, C34.19, C34.21-C34.25, C34.29, C34.31-C34.35, C34.39 C34.81-C34.85, C34.89, C34.91-C34.95, C34.99

mSNOMED-codes for NSCLC: M80703, M81403, M80123, M85603, M80103

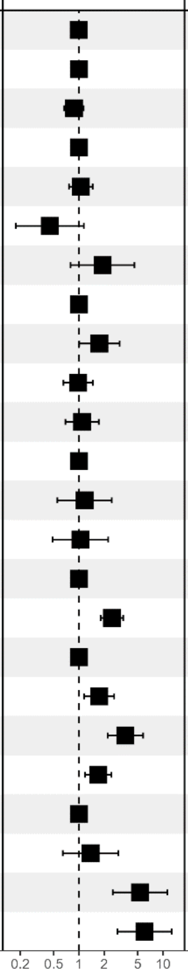

Variable		N	Hazard ratio		p
**Age**		379		0.99 (0.98, 1.01)	0.390
**Sex**	Male	188		Reference	
	Female	191		0.87 (0.67, 1.14)	0.306
**CCI**	0	263		Reference	
	1-2	100		1.05 (0.76, 1.45)	0.767
	3-4	10		0.45 (0.18, 1.14)	0.092
	5-	6		1.90 (0.79, 4.57)	0.150
**Molecular alterations**	Other driver mutation	66		Reference	
	KRAS G12C	35		1.75 (1.01, 3.04)	0.048
	No driver mutation	205		0.98 (0.65, 1.46)	0.907
	Other KRAS	73		1.09 (0.69, 1.72)	0.718
**Smoking**	Never smoked	15		Reference	
	Previous smoker	165		1.16 (0.55, 2.45)	0.690
	Smoker	199		1.04 (0.49, 2.23)	0.920
**Treated**	yes	212		Reference	
	no	167		2.46 (1.81, 3.36)	<0.001
**Performance status**	0-1	171		Reference	
	2	51		1.74 (1.15, 2.63)	0.009
	3-4	35		3.56 (2.19, 5.80)	<0.001
	Unknown	122		1.69 (1.18, 2.43)	0.004
**Stage**	I-II	26		Reference	
	III	108		1.37 (0.64, 2.94)	0.414
	IV	125		5.33 (2.52, 11.25)	<0.001
	Unknown	120		6.02 (2.87, 12.63)	<0.001

Supplemental Figure S1. Cox model for OS including also treatment status, performance status, and tumor stage as covariates. The proportional hazard assumptions were violated for these additional covariates and thus, the hazard ratios represent an average risk over time.

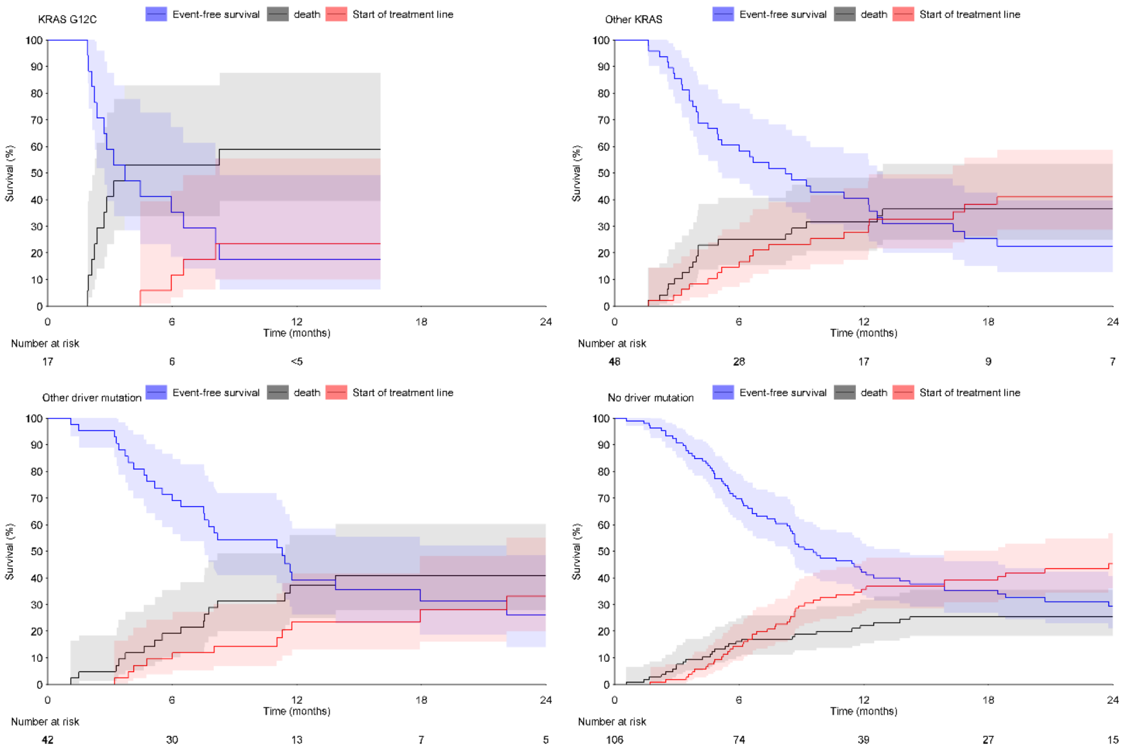

Supplemental Figure S2. Time to next treatment of the first treatment line by *KRAS* subgroups using a competing risk model (death as competing risk). Event-free survival (blue curve) indicates the composite event of start of the next treatment line and death. Median TTNT from the event-free survival: *KRAS* G12C 2.8 months (95% CI 1.2-5.2), other *KRAS* 6.9 months (95% CI 3.4-10.4), other driver mutation 10.1 months (95% CI 5.7-16.5), no driver mutations 7.9 months (95% CI 6.3-11.1).
