# Supplementary figures and images for: Real-World Evidence Study of Patients with KRAS-Mutated NSCLC in Finland
Source: Curr Oncol. 2024 May 11;31(5):2700–12. doi: 10.3390/curroncol31050205 (PMC11120216; doi:10.3390/curroncol31050205)

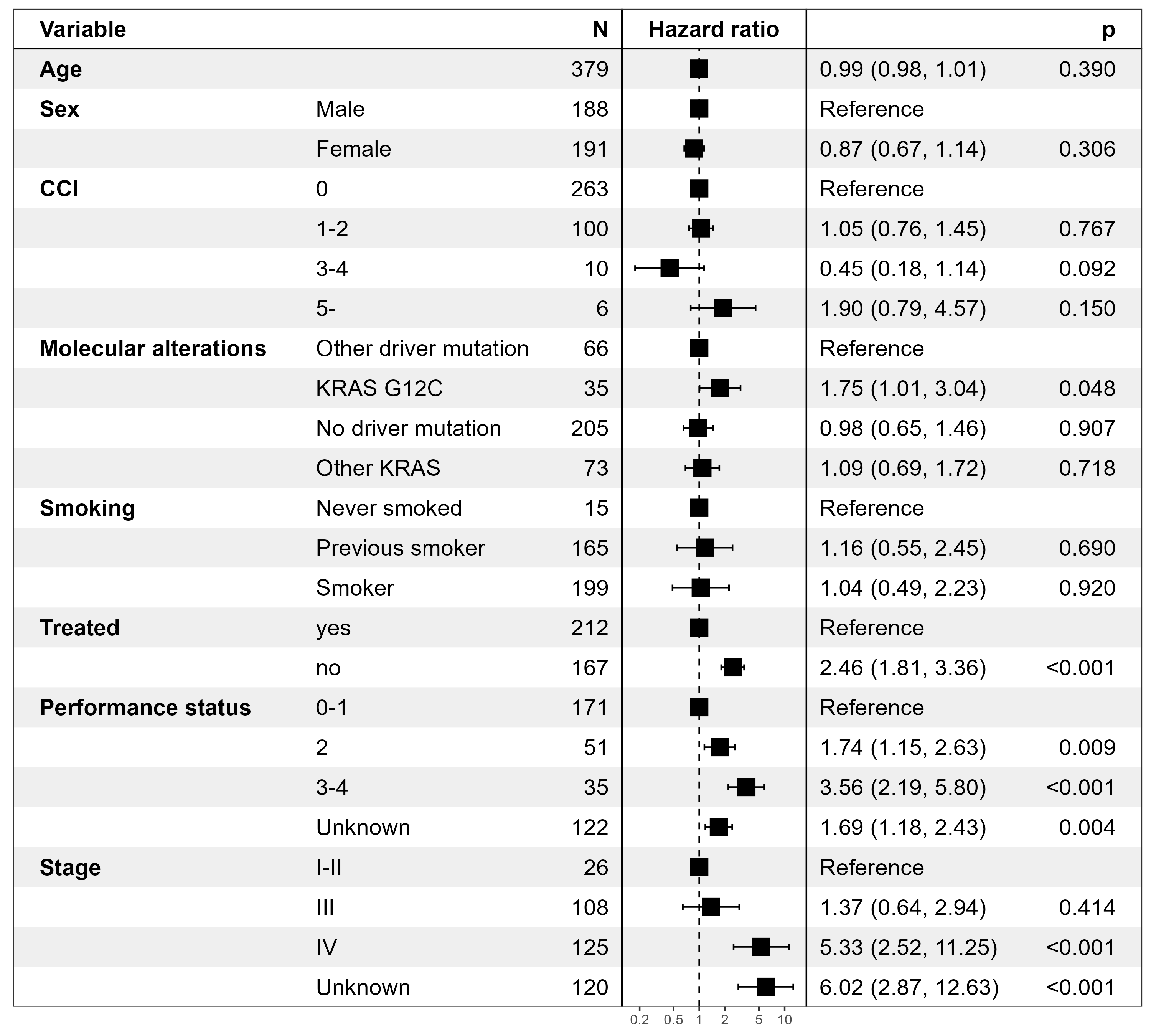

Supplement: Supplementary file 1 [file curroncol-31-00205-s001.zip › Supplementary Figure S1.tiff]

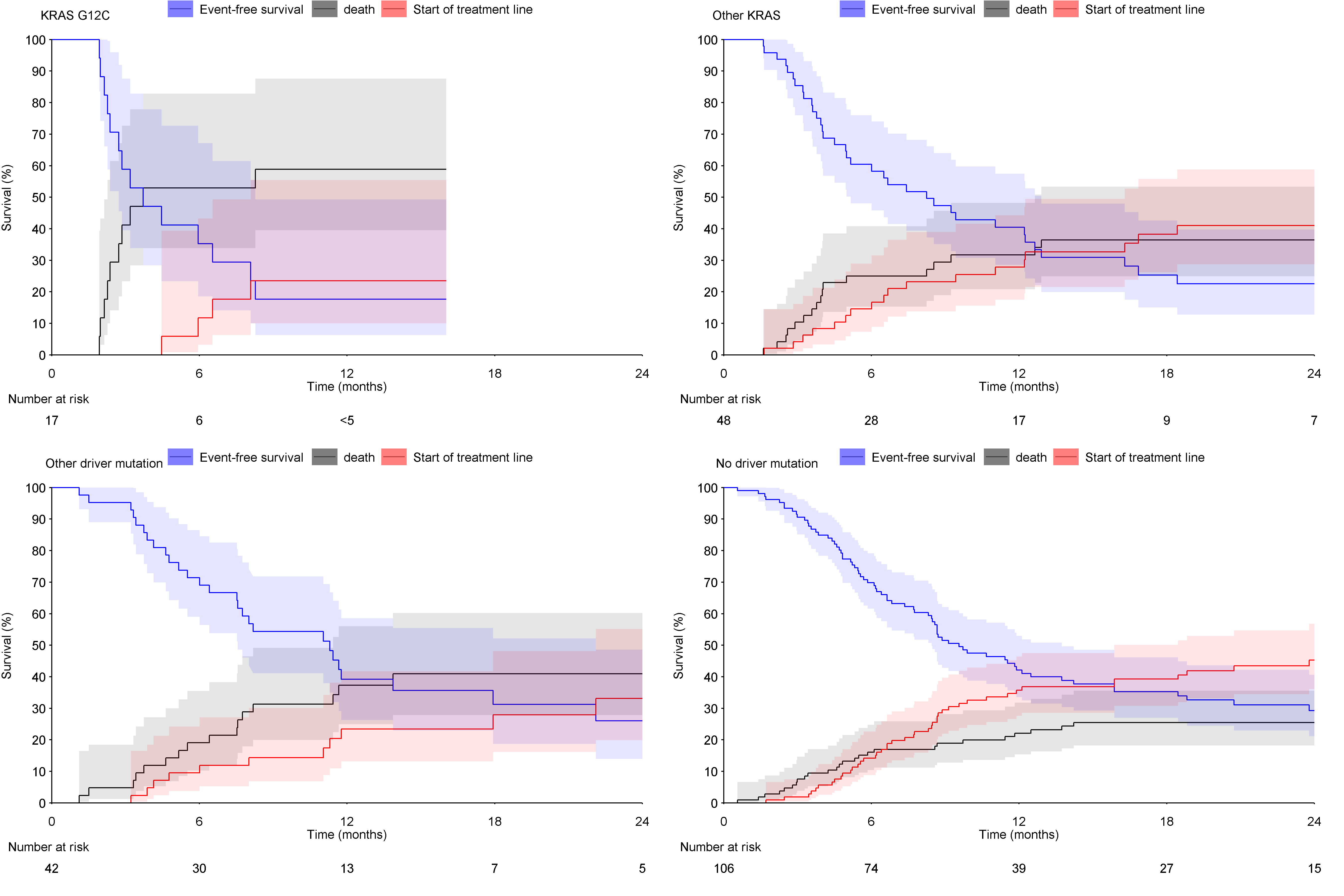

Supplement: Supplementary file 1 [file curroncol-31-00205-s001.zip › Supplementary Figure S2.tiff]
